# Science, conflicts of interest and ethics

**DOI:** 10.1590/0100-6991e-20213256EDIT01

**Published:** 2022-02-18

**Authors:** MARIA ISABEL TOULSON DAVISSON CORREIA

**Affiliations:** 1 - Universidade Federal de Minas Gerais, Cirurgia - Belo Horizonte - Minas Gerais - Brasil

## EDITORIAL

The Journal of the Brazilian College of Surgeons has required, since the second semester of 2021, that all authors of any submitted manuscript, mandatorily, fill in and sign the conflict of interest form. This agrees with the suggested demands by the International Committee of Medical Editors^1^ and aims to minimize the negative impact that potential individual or collective conflict of interest may impose on the community. 

Questionings such as “if I tell you that all authors have participated in the paper, then this is the truth, so why are you challenging me for this aspect?” or statements such as “considering everything I have done for the surgical practice in this country, this deserves to be highlighted and published” were among the many messages received by this editor. The idea that name(s) or institution(s) behind a paper is enough to guarantee the publication was firmly rejected throughout this period, and one may imagine the consequences of such behavior. 

Conflict of interest exposes the community and the patients to huge risks. A conflict of interest is by definition “a group of conditions for which the professional judgment related to a primary interest (ex. the well-being of a patient or the validity of a research question) tends to be influenced by a secondary interest (ex. financial profit). However, it is important to highlight that such secondary interest is not always of economic origin but is often driven by a passion for knowledge or personal ambition^2^. When medical societies are involved, the primary interest should be targeted to the mandatory and solid basic principles of ethics. After all, Medicine is essentially linked to the practice of moral and ethical actions. Therefore, when physicians join a society, they expect these entities will reach their expectations related to professional defense, educational, and research activities^3^. The global group of interests aims to promote the “wellness” to those at the front line of the provided services: the patient! However, such moto has commonly been overpassed by political, commercial, and economic agendas to the detriment of the fundamental principle of ethical obligations^3^. 

Distinct actions against conflicts of interest data back to 1907. In that year, George H. Simmons published in the Journal of the American Medical Association (JAMA) the first interpretation of the topic, challenging the passivity of the physicians of the time in the face of the deluge of medications produced by the industry, many of them without the approval of pharmacists, and whose “creators” used pervasive marketing to influence the healthcare professionals, especially, physicians^4^. According to him, “the business associated with new medications has promoted great advances in the scientific method, raising an exaggerated optimism not supported by the facts - an optimism more fatal than the most radical therapeutic nihilism. But, above all and worst, such practices destroyed the literature by mocking the medical journals and challenging the quality of the books”^4^. 

Science and the scientific literature, challenged since then, have quickly and tremendously grown to reach such demands, but they balance in a slim line of ethical principles. As a scientist and educator myself, I thank the evolution to a more civilized world, in which bonfires are not the end of those who challenge the paradigms and defend the ethics and morals among peers. Otherwise, as a common practice, 500 years ago, this person would have ended in one such bonfire, just because it questions and challenges everything, which was at that time understood as an act of witchery. The Italian monk, Giordano Bruno, was condemned to death and burnt because she believed and defended the free-thinking of philosophy and science. Galileo Galilei, for very little, did not have a similar end, but to get away from such condemnation, he had to refute in public that he did not support Copernicus’ ideas^5^. Thousands of women faced all these situations and many more, just because they dared to speak up, and of course, they ended up burnt in bonfires. We have evolved but far from reaching ethics! 

The concept of ethics was initially proposed by Aristoteles to discuss the daily principles of “ethike theoria” as well as to study and offer criteria to assess human behavior. Therefore, ethics has been one of the big western philosophy themes, mainly when individual and social values are at stake, and the relation and hierarchy of society are debated. Currently, the meaning of ethics mingles with those of moral, a word whose origins are Latin “mos, moris,” which also means a habit or behavior but more associated with individual principles. So, these two - ethics and moral - should be thoroughly discussed by scientific societies, pillars of knowledge translation, but that daily face enormous challenges due to the advances of Medicine and all the industry behind it. Nonetheless, it is inevitable to acknowledge the relevant role of the industry in the educational and scientific process, especially in countries where there is no government support for these practices. However, “Science san conscience et la ruine l’âme” (Rabelais, em Pantagruel)^5^, this is to say, science must be submissive to moral to prevent exaggeration. The freedom to be able to speak and express oneself, as well as the freedom not to fear, are two principles that should guide the human being, well-covered by the past United States President - Franklin D. Roosevelt in the State of the Union speech delivered on January, the 6^th^, 1941^6^. Such principles were pillars that later supported the writing of the Human Rights Declaration, signed in 1948. In 2005, the then general secretary of the United Nations, Kofi Anan, highlighted the relevancy of supporting the right to healthcare as an integral part of Human Rights. And, since science supports better healthcare practice, this is also a Human Right ^7^ ^,^ ^8^. 

The reasons that justify science as a Human Right are straightforward related to the principle of empowerment of the individual and the community and the improvement of the quality of life of all^8^. Thus, the practice of good science without ethics and moral is against the benefits associated with the best healthcare services, the latter a fundamental right that every human being should be entitled to^9^. Moreover, bad science is against the 29 topics covered in the 2018 publication by the Economic, Social, and Cultural forum of the United Nations on the right to benefit from science and the intellectual property of knowledge translation^8^. Therefore, science translation must be free from political control or any other conflict of interest. Ignoring such is an attack on ethics and moral! 

Thèrese Murphy^10^ defends the utmost important role of the international institutions considered “Palaces of Hope,” even though, in reality, they act precariously and far from those who need their protection. According to her, these institutions are weak when facing power. They are also poorly focused on excused and mudded support due to the lack of reforms, while populist and authoritarian governments are on the rise. 

Finally, I should thank all those who have contributed to my trajectory, without naming any not to incur mistakes. Thank you so much! See you!
